# Neural mechanisms in remote ischaemic conditioning in the heart and brain: mechanistic and translational aspects

**DOI:** 10.1007/s00395-018-0684-z

**Published:** 2018-06-01

**Authors:** Marina V. Basalay, Sean M. Davidson, Andrey V. Gourine, Derek M. Yellon

**Affiliations:** 1 The Hatter Cardiovascular Institute, University College London, 67 Chenies Mews, London, WC1E 6HX UK; 2 Department of Cardiology, Karolinska University Hospital, 171 76 Stockholm, Sweden

**Keywords:** Remote ischaemic conditioning, Cardioprotection, Neuroprotection

## Abstract

Remote ischaemic conditioning (RIC) is a promising method of cardioprotection, with numerous clinical studies having demonstrated its ability to reduce myocardial infarct size and improve prognosis. On the other hand, there are several clinical trials, in particular those conducted in the setting of elective cardiac surgery, that have failed to show any benefit of RIC. These contradictory data indicate that there is insufficient understanding of the mechanisms underlying RIC. RIC is now known to signal indiscriminately, protecting not only the heart, but also other organs. In particular, experimental studies have demonstrated that it is able to reduce infarct size in an acute ischaemic stroke model. However, the mechanisms underlying RIC-induced neuroprotection are even less well understood than for cardioprotection. The existence of bidirectional feedback interactions between the heart and the brain suggests that the mechanisms of RIC-induced neuroprotection and cardioprotection should be studied as a whole. This review, therefore, addresses the topic of the *neural* component of the RIC mechanism.

## Introduction

**Fig. 1:**
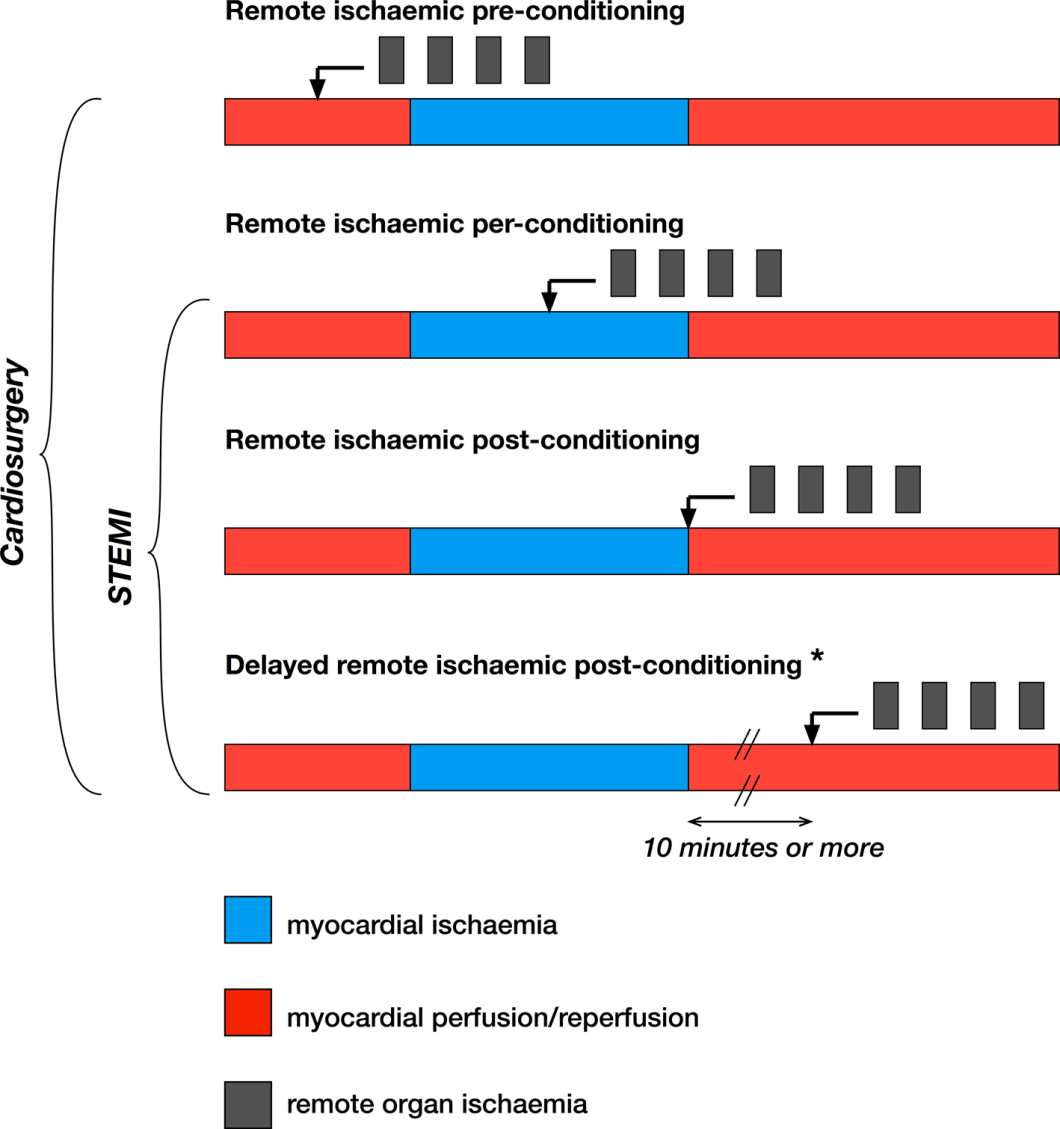
Schematic time lines representing the concepts of remote ischaemic pre-, per-, post- and delayed postconditioning, and the clinical scenarios in which they may be relevant. *STEMI* ST-elevated myocardial infarction. Asterisk—the evidence for the existence of the delayed remote ischaemic postconditioning phenomenon is currently limited having only been demonstrated in one experimental study

It is almost 25 years since the phenomenon of remote ischaemic *pre*conditioning (RIPre) was discovered by Przyklenk et al. [106]. They demonstrated in a canine model that a series of brief, alternating episodes of ischaemia and reperfusion, applied to one area of the left ventricle (LV) myocardium, protected a remote myocardial territory of the same heart from a subsequent prolonged ischaemic insult followed by reperfusion, limiting infarct size by almost 70%. In the same year, McClanahan et al. [94] achieved a similar infarct-limiting effect in rabbits by applying a single 10-min episode of ischaemia to a kidney prior to 30-min occlusion of the left anterior-descending coronary artery. However, since the RIPre stimulus must, by definition, be applied before the onset of ischaemia, it is clear that the only possible clinical niche for the RIPre phenomenon in cardioprotection could be in the setting of either elective cardiac surgery or elective percutaneous coronary intervention (PCI)—scenarios when the onset and the duration of ischaemia are carefully controlled (Fig. 1).

Twelve years after the discovery of RIPre, it was shown in a rat model that remote ischaemic conditioning (RIC) could be applied to a limb *during* myocardial ischaemia, and reduce infarct size by 50% [77]. This phenomenon became known as remote ischaemic *pre*conditioning (RIPer). The next step in the study of RIC came with the demonstration by Andreka et al., that RIC could be successfully administered *simultaneously with the onset of myocardial reperfusion*—a phenomenon named as remote ischaemic *post*conditioning (RIPost) [1]. Taking this one step further, Basalay et al. demonstrated in a rat model of acute myocardial infarction that RIPost *applied after a 10*-*min delay following the onset of reperfusion* was still able to reduce infarct size similarly to RIPre [5]. Notably, the results included in this study were obtained in three research centres, using identical protocols [5]. The success of this procedure, termed ‘delayed RIPost’, was unexpected, in view of the prevailing hypothesis that the majority of myocardial reperfusion cell death is due to the opening of mitochondrial permeability transition pores (mPTP), thought to occur during the first few minutes of reperfusion [29, 46]. However, a series of studies demonstrated implication of non-mPTP/necrosis mechanisms in myocardial death [59] including pyroptosis [147], necroptosis [12, 127], and, more controversially, apoptosis [67]. We appreciate that the Basalay study is the only one to-date reporting the benefit of delayed RIPost [5], and is, therefore, still awaiting independent confirmation. Nonetheless, the clinical potential of this cardioprotective phenomenon can be significant, as it potentially broadens the time frame during which myocardial injury can be prevented. Moreover, the possibility to protect myocardium beyond the first few minutes of reperfusion has also been supported by studies from the groups of Ovize, Marbán and others [4, 27, 38, 71, 116], which have demonstrated cardioprotection with interventions applied up to the 20th min (rats, isolated rat hearts) [27, 38], 30th min (minipigs, mice) [71, 116] or 45th min (rats) [4] of myocardial reperfusion.

Though RIPre first emerged as a laboratory phenomenon, the cardioprotective potential of RIC strategies has subsequently drawn the attention of numerous clinical research groups [15, 28, 31, 36, 40, 82, 96, 105, 133, 135, 141]. In contrast to the repeated failures to translate cardioprotection from animal studies to clinical practice [42, 51, 59], RIC still appears to be a promising candidate for clinical use based both on the large number of successful experimental studies [17] and the fact that it satisfied all the recommendations for preclinical trials on cardioprotection [53, 56, 59, 86, 87].

The majority of clinical studies to date have demonstrated the infarct-limiting effect of RIC [15, 21, 28, 36, 105, 141] and improved outcome [40, 125] as a result of this procedure in *patients with STEMI*. The one exception is a recent study with neutral results, however, no long-term outcome data are currently available from this study [135]. In the setting of *elective cardiac surgery*, a single-centre trial involving 329 patients with isoflurane, used for the maintenance of anesthesia, suggested that RIPre provided perioperative myocardial protection [133] and improved the prognosis over 1.54 years [133] and 5 years, respectively [81]. Another small-scale clinical study on patients undergoing elective aortic valve replacement, demonstrated that RIPre reduced myocardial injury if applied on a background of sevoflurane, but not propofol anesthesia [9, 10]. Two, large, multicentre clinical trials on cardiac surgery patients using propofol for anesthesia [31, 96], and one small clinical study with perioperative anesthesia restricted to sevoflurane and fentanyl [99] failed to show any benefits of using RIPre. Bearing in mind the success of the previous (proof of concept) clinical trials both in STEMI patients and in patients undergoing cardiac surgery, these neutral results indicate the gaps in the knowledge of the mechanisms underlying the RIC phenomena, as well as lack of the differences between the mechanisms of myocardial ischaemia/reperfusion injury in these two clinical settings. It is, therefore, imperative to investigate the mechanism of RIC to facilitate the translation of this simple, non-invasive, low-cost intervention into patient benefit. Two large-scale multicentre clinical trials on STEMI patients are ongoing (NCT02342522; NCT01857414). Their results, due in 2019, will shed more light on the effects and possible confounding factors of cardioprotection by RIC.

## The mechanisms of cardioprotection by remote ischaemic conditioning with regard to translational aspects

After the discovery of RIPre, two main competing hypotheses evolved regarding the mechanism of signal transduction from the preconditioned limb to the protected heart, one called the ‘humoral hypothesis’ and the other—the ‘neural hypothesis’. The humoral hypothesis is based on the fact that RIC-induced cardioprotection can be transferred to a naïve animal via whole blood transfusion [32]. Conversely, Gho et al. demonstrated that the neural blocker, hexamethonium, abolished cardioprotection by brief mesenteric artery occlusion [43], suggesting the important role for the autonomic ganglia in RIPre. However, in a later study by Weinbrenner et al., hexamethonium failed to inhibit the protection afforded by RIPre, performed as infrarenal occlusion of the aorta [140]. This result, as well as the necessity for a reperfusion period interspaced between the RIPre and myocardial ischaemia, during which time the humoral factor could travel to the heart, were viewed as reasons to exclude the hypothesis of a neuronal signal transmission from the remote area to the heart [140].

**Fig. 2:**
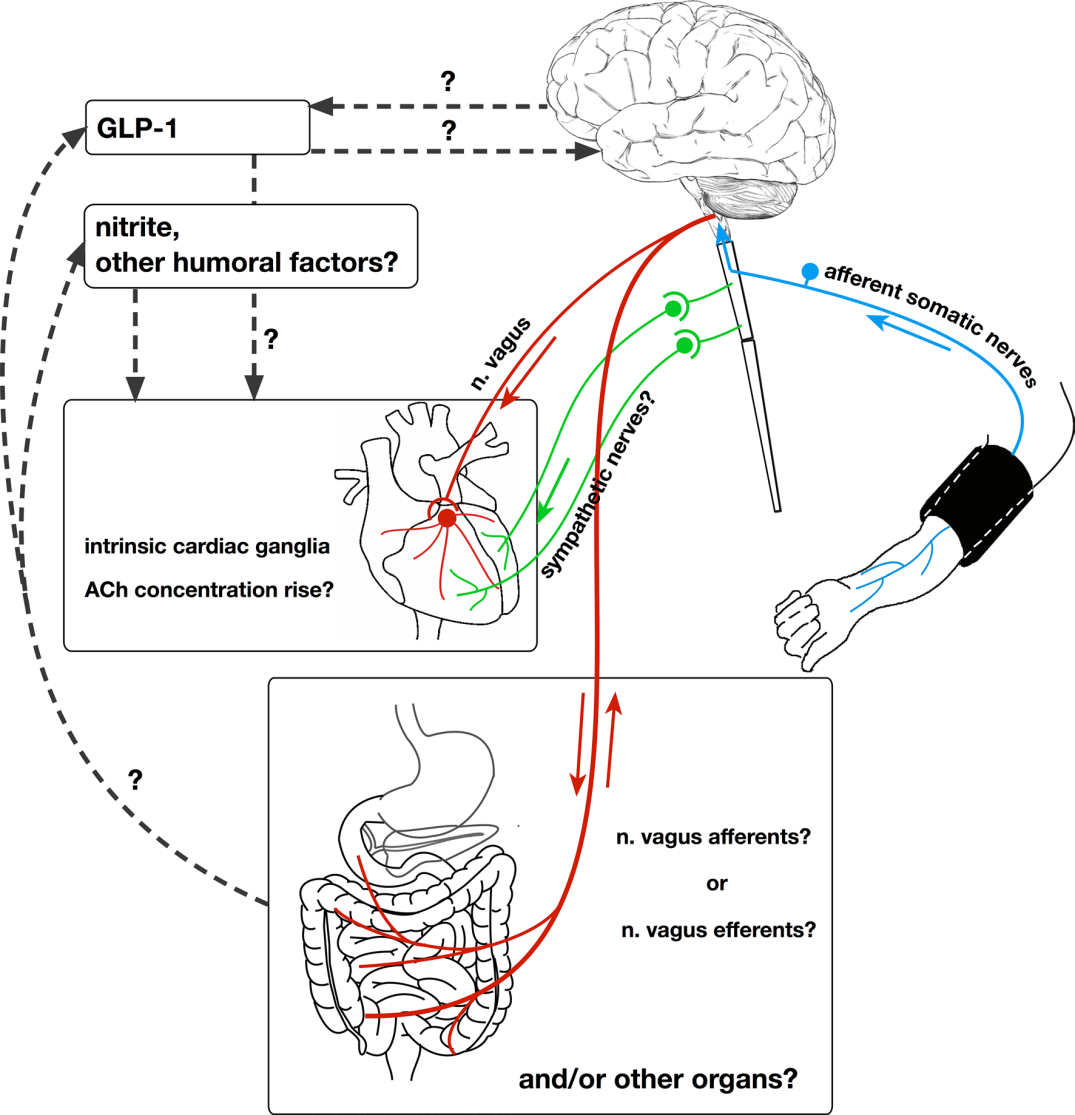
Neural mechanisms of remote ischaemic preconditioning. A diagram of connections (both known and controversial) between the neural and humoral mechanisms of remote ischaemic preconditioning of the heart and the brain. The core of this mechanism is the ‘remote preconditioning reflex’ comprising afferent somatic nerves, integration centre/centres in the central neural system, and efferent vagal nerves, innervating the heart and other organs, specifically, the intestine. However, it is not certain whether the increase in ACh concentration in the myocardium mediates the cardioprotective effects of remote preconditioning. It is also unclear which of the abdominal n. vagus fibres—afferent or efferent—are involved in this phenomenon. The known humoral factors that have the closest relationship with neural mechanisms are GLP-1 and nitrite. However, the source of their release in response to remote ischaemic stimulus is not clear. *GLP-1* glucagon-like peptide-1, *ACh* acetylcholine

In 2010, Gourine et al. proposed the existence of a ‘remote preconditioning reflex’—a neural pathway of cardioprotection which is recruited by RIPre and protects the heart against ischaemia and reperfusion injury [44] (Fig. 2). This idea was supported by the data indicating the presence of all the components necessary for the realization of a classical reflex in the pathway mediating cardioprotection by RIPer: sensory (afferent) C-fiber neurons, an integration centre in the central nervous system, and motor (efferent) vagal neurons. In this regard, it has been demonstrated in a rat model of myocardial ischaemia/reperfusion injury, that cardioprotection by RIPre is completely abolished by denervation of the limbs performed as either mechanical transection of nerves [5, 130] or blocking afferent C-fibers by neonatal capsaicin treatment [5]. Similarly, stimulation of C-fiber afferents by topical capsaicin application limits myocardial injury, mimicking the effect of RIPre [5, 112]. It has been shown that calcitonin gene-related peptide (CGRP)—an important mediator of sensory neurons—mediates cardioprotection by RIPre [142]. Abrogation of protection by RIC with spinal cord transection at T7–T10 level [34], or with intrathecal spinal opioid receptor blockade [143], confirmed the involvement of the neural afferent pathway. The effector arm of the RIPer reflex appears to involve parasympathetic innervation. In the studies by Basalay et al., bilateral vagotomy in a rat model abolished cardioprotection induced by either RIPre [5, 6] or RIPer [6]. Mastitskaya et al. went further and directly inhibited parasympathetic motor neurons which are known to be located in two distinct brainstem sites [93]. This resulted in complete abolishment of the infarct-limiting effect of RIPre [93]. Notably, this study was the first to provide direct evidence for the involvement of vagal efferents in RIPre-mediated cardioprotection. It is noteworthy that bilateral vagotomy in the Basalay et al. study did not attenuate the infarct-limiting effect of delayed RIPost [5], which suggested that the mechanisms underlying these two phenomena may be distinct. Surprisingly, in a recent study by Buchholz et al., vagal stimulation in mice reduced myocardial infarct size when applied either before ischaemia or at the onset of reperfusion [18]. However, this study also revealed the difference in cardioprotective mechanisms of these two time-periods of vagal stimulation: the first activated the Akt/GSK-3β muscarinic pathway, while the second activated α7nAChR and JAK-2, independently of the cholinergic anti-inflammatory pathway [18]. The results of these studies [5, 18] raise the possibility that additive benefit could be achieved by combining different types of RIC. A recent study by Kleinbongard et al. confirms the neuronal transfer of the protective signal during RIC [78]. In this study, RIC was demonstrated to attenuate ischaemia-induced ST-segment elevation on the ECG during ongoing coronary artery occlusion [78].

Yellon’s group were the first to demonstrate the involvement of both neural and humoral pathways in RIPre [89]. They found that in an RIPre model of femoral artery occlusion in mice, the absence of venous blood return from the preconditioned limb, or combined femoral and sciatic nerve resection, completely abolished the infarct-limiting effect. Moreover, resection of only one nerve—either femoral or sciatic—only partially abolished this effect [89]. In an elegant study, Jensen et al. confirmed the requirement for both the neural and humoral pathways in RIPre-mediated cardioprotection by testing whether human plasma dialysate obtained after RIC could reduce infarct size and improve hemodynamic recovery in isolated rabbit hearts [64]. They showed that the plasma dialysate was protective if obtained from healthy subjects or diabetic subjects without peripheral neuropathy, but when obtained from diabetic patients with neuropathy it was not protective. These findings further indicate that the mechanism of a humoral factor release involves neural pathways [64].

Because the initial studies investigating the role of the autonomic ganglia in RIPre-mediated cardioprotection had been controversial [43, 140], this question was re-visited by Yellon’s group in 2016 [102, 103]. They demonstrated that plasma dialysate obtained from RIPre-treated rats reduced infarct size in naïve isolated hearts subjected to ischaemia and reperfusion. However, the plasma was no longer cardioprotective if collected from vagotomised animals, or if the ganglionic blocker hexamethonium or muscarinic antagonist atropine were applied to isolated hearts [103]. This led to the conclusion that release of a protective factor following RIC is dependent on prior activation of the vagus nerve. In addition, the factor appears to induce cardioprotection via recruitment of intrinsic cardiac ganglia. Their subsequent study, demonstrating that coronary effluent from a preconditioned isolated heart could protect another isolated heart, but not isolated cardiomyocytes, also contributed to the hypothesis of the importance of intrinsic cardiac ganglia in the mechanisms of direct ischaemic preconditioning [102].

Since the first demonstration of the possibility to transfer ischaemic preconditioning cardioprotection with blood [32], the attention of several research groups has focused on identifying the humoral factor responsible for RIPre. Several candidate humoral factors of RIC have been proposed, including stromal cell-derived factor-1 [30], nitrite/nitric oxide [110], interleukin-1α [41], interleukin-10 [19], microRNA-144 [88], apolipoprotein A-I [57], alpha-ketoglutarate-dependent dioxygenase Egln1 [101] and glucagon-like peptide-1 (GLP-1) [7, 8]. However, for the purpose of this review, we focus on two of the proposed humoral mediators that are most relevant to the ‘neural hypothesis’ of RIC, namely nitrite/nitric oxide [110] and GLP-1 [7, 8].

Using an in vivo mouse model, Rassaf et al. demonstrated that shear stress-dependent stimulation of endothelial nitric oxide (NO) synthase within a femoral artery by RIPre yields a substantial release of NO. This NO is subsequently oxidized to nitrite and transferred humorally to the myocardium, where it reduces infarct size caused following 30-min myocardial ischaemia and 24-h reperfusion [110]. In addition, a series of experiments in which plasma from healthy volunteers subjected to RIPre was perfused through isolated mouse hearts, identified plasma nitrite as the cardioprotective agent [110]. Similarly, in a recent study by Hauerslev et al., scavengers of NO attenuated RIPre-induced protection in rat hearts [47]. Previously, it had been shown that either stimulation of cervical vagal nerves or perfusion with acetylcholine is associated with NO release in an isolated rabbit heart via the neuronal isoform of NO synthase [16]. Interestingly, in an earlier study, bilateral vagal nerve stimulation in rabbits was followed by an increase in nitrite formation at the level of the stomach and the colon [62]. In terms of planning future clinical studies on the effect of RIC, it is important to keep in mind that exogenous NO [98] and nitroglycerin [35, 50, 148, 155] have been shown to be able to reduce infarct size, and may therefore, potentially interfere with RIC.

The results obtained by Mastitskaya et al. [92] suggest that visceral organs, innervated by the posterior gastric branch of the vagus nerve, are the likely source of a humoral factor (or factors) of RIC cardioprotection. This finding is interesting as it provides the first potential *mechanism uniting the ‘neural’ and ‘humoral’ hypotheses of RIC*. Apart from nitric oxide [62], GLP-1 appears to be the most likely candidate for this role. Its release from the L-cells of the intestine is modulated by vagal efferent (motor) activity [58, 115] and there is also evidence that GLP-1 may interact with vagal sensory fibers innervating the viscera [58]. The molecular weight of GLP-1 is 3.3 kDa thereby satisfying the key criteria of the humoral preconditioning factor (including molecular weight of less than 8 kDa), suggested by Lang and colleagues on the basis of the proteomic analysis of blood samples obtained from experimental animals receiving the RIPre stimulus [85]. Studies conducted in animal models by the Yellon group [13, 14], and subsequently by others [111], demonstrated potent cardioprotection by GLP-1 receptor (GLP-1R) activation. The efficacy of GLP-1R agonists in limiting infarct size has also been shown in clinical trials on STEMI patients [90, 144]. Later, Basalay et al. showed that GLP-1 mediates cardioprotection by RIC, and demonstrated that cardioprotection induced by GLP-1R activation is mediated by a mechanism involving M3 muscarinic receptors [7, 8]. The location of these M3 muscarinic receptors, mediating the infarct-limiting effect of RIPre, is not yet established.

The main extracellular signaling molecules that have been shown to be involved in cardioprotection by RIPre include opioids, bradykinin and adenosine [79]. Opioid peptides can be secreted from cardiac nerves or produced in the cardiomyocytes themselves [107]. Shimizu et al. demonstrated that the protective effect of dialysate from a donor limb subjected to RIC, on isolated cardiomyocytes is blocked by pre-treatment of the cardiomyocytes with the opiate receptor blocker naloxone [122]. These results suggest that opioid receptors involved in RIC are located in cardiomyocytes and not connected with neural activation. On the other hand, in another study, hexamethonium abolished the protection provided by intramesenteric bradykinin infusion [119]. Regarding bradykinin, it is worth mentioning that it is degraded rapidly by angiotensin-converting enzyme (ACE) [136], while ACE inhibitors and angiotensin-1 receptor antagonists [63] mimic its cardioprotective effects. The role of adenosine in RIC cardioprotection is still controversial, being confirmed in a mouse model [120], but not a porcine model [48].

Analysis of the concentrations of metabolites in plasma samples of patients subjected to RIC revealed an increase in glycine concentration following RIC [23]. This is relevant to the current review, as glycine is known to be an inhibitory neurotransmitter in the central nervous system, and specifically, in parasympathetic nuclei [22, 131, 132]. In the above-mentioned study, the injection of glycine mimicked the protective effects of RIC in rats [23]. García-Dorado’s group demonstrated that glycine exerts cross-species cardioprotection against infarction through glycine receptor activation [66]. The potential involvement of glycine signalling in RIC-induced cardioprotection should be borne in mind when planning clinical trials, as certain drugs, such as Ketamine [137], can inhibit glycine neurotransmission to cardiac vagal neurons.

The importance of understanding the mechanism of RIC cardioprotection is clearly demonstrated by the neutral results of two recent multicentre trials—ERICCA and RIPHeart, in which the majority of the patients were given propofol as part of the anesthesia protocol [31, 96]. Importantly, it had previously been shown that propofol inhibits neurotransmission to cardiac vagal neurons in the nucleus ambiguous [138]. On the other hand, propofol had been shown to cause the enhanced nitrite production in cultured myocytes [146], whereas nitrite is known as one of the humoral mediators of RIPre [110]. The results of small-scale clinical trials completed in 2012 and 2014 confirmed the importance of the anesthetic regime, showing that RIPre during isoflurane but not during propofol anesthesia decreased myocardial damage in patients undergoing CABG surgery [82] or elective aortic valve replacement [9, 10]. A recent experimental study also clearly demonstrated that propofol, but not sevoflurane, abolished the infarct-limiting effect of RIPre in rats in vivo [11]. In a more recent single-centre study with a neutral effect of RIC in CABG patients, propofol was purposefully avoided [99]. However, in this study, the anesthetic protocol included fentanyl, which has been shown to inhibit neurotransmission to cardiac vagal neurons in the nucleus ambiguous [45]. It could also be argued, however, that fentanyl or sufentanil did not abolish RIC-induced cardioprotection in other clinical studies [9, 133]. Although there are multiple possible reasons for the lack of protection in these studies, as has been extensively discussed [25, 39, 55], the suppressed vagal system is likely to have contributed.

The effect of an anesthetic protocol on RIC efficacy was revealed in a Bayesian network meta-analysis of 55 randomized trials, which included 6921 patients undergoing cardiac surgery. The use of volatile agents and the combination of volatile agents with remote preconditioning were found to be associated with a reduction in mortality at the longest follow-up time available, when compared to total intravenous anesthesia [151]. Furthermore, it was observed that the combination of RIC with volatile agents was associated with a reduction in mortality when compared to RIC with total intravenous anesthesia [151].

## Unresolved questions and discrepancies on the mechanisms of cardioprotection by remote ischaemic conditioning

*Although the degree of cardioprotection conferred by RIPre and delayed RIPost is similar, the underlying mechanisms seem to be distinct* [5]. In the Basalay et al.’s study (2012), the infarct-limiting effect of delayed RIPost was not affected by either vagotomy or peripheral denervation, as opposed to the effect of RIPre [5]. As peripheral or especially parasympathetic denervation, can potentially represent real clinical situations with patients suffering from peripheral polyneuropathies of different etiologies, or exposed to drugs with vagolytic effects, these results allow us to predict a lack of cardioprotective benefit from RIPre in these cohorts of patients. On the other hand, *delayed RIPost could potentially be more effective* in the same patients, being less susceptible to innervation impairments, though no clinical trials demonstrating this have been completed so far.

The other *missing part in our knowledge is the mechanism of vagally mediated RIPre cardioprotection*. The feasibility of vagally mediated cardioprotection (i.e., RIPre) in a left ventricle, which is known to be only sparsely innervated, can be explained by the observation that there is a local acetylcholine (ACh) synthesis system in the myocardium [37, 69, 109]. This system is positively modulated by cholinergic stimuli [69]. Conversely, atropine reduces the basal ACh content [69]. It has been shown that either bilateral vagotomy [5] or direct inhibition of the parasympathetic motor neurons in the central nervous system [93], or atropine treatment [34] abolishes the infarct-limiting effect of RIPre. Yet, it is not clear whether it is the increase of acetylcholine concentration that mediates the cardioprotective effect of RIC. There are a number of experimental observations relevant to the assumption that it is indeed acetylcholine which mediates RIPre. First, in support of this hypothesis, the hearts from transgenic mice overexpressing choline acetyltransferase (ChAT)—the main ACh-synthesizing enzyme in cardiomyocytes—are more resistant to ischaemia–reperfusion injury than wild-type hearts [70]. And second, RIC increases both ChAT expression and ACh content in mice hearts [68, 100]. However, there are also experimental data that contradict this hypothesis. In this regard, it has been shown that acute myocardial ischaemia provoked an increase of ACh concentration in an ischaemic myocardium, and vagotomy did not affect this increase [76], while on the other hand, vagotomy abolished the infarct-limiting effect of RIPre [5]. This indicates that the loss of RIPre-mediated cardioprotection in vagotomised rats [5] might be caused by some other factors rather than a decrease in ACh concentration. Another indirect argument suggesting that ACh concentration may not be of critical importance for ischaemic preconditioning is that vagotomy abolishes the myocardial interstitial ACh release induced by brief myocardial ischaemia [75]. On the other hand, bilateral vagotomy does not attenuate the infarct-limiting effect of direct ischaemic preconditioning [5], which indicates that ACh concentration in the myocardium does not correlate with the degree of cardioprotection. Currently, there are no data revealing whether RIPre causes further increase of ACh concentration in the ischaemic myocardium in comparison to the myocardial ischaemia itself, and whether vagotomy prevents this additional increase. The answer to this question is even more difficult, as the degree of ACh increase during myocardial ischaemia varies from study to study, depending on the animal species, samples and techniques used, as well as on the duration of the ischaemic episode [74, 76, 102].

Despite the lack of experimental data that would unequivocally answer the question of myocardial ACh concentration in RIC-mediated cardioprotection, there are several observations that may shed some light on this aspect. In this respect, it was demonstrated that *cardiomyocytes of adult, but not neonatal rats, are able to synthesize, transport and excrete ACh in the heart* [109]. The expression level of ChAT and the amount of ACh excreted were *also significantly downregulated in cardiomyocytes of old animals* [109]. If ACh concentration in myocardium determines the potency of protection by RIC, we may expect that RIC would be less effective in children and aged people. Indeed, RIPre was actually shown to impair ventricular function and increase infarct size in an isolated neonatal rabbit heart [118]. An earlier, small-scale clinical study had demonstrated myocardial-protective effects of RIPre in children [26]. However, in a more recent clinical trial performed on 299 children (aged neonate to 17 years), RIPre was not associated with important improvements in clinical outcomes and physiological markers after cardiac surgery [95]. It should be noted, however, that there was no standardized protocol of anesthesia in this study, and more than half the patients were exposed to propofol [95]. The effect of RIC in elderly patients (> 70 years) was investigated in the CONDI trial, and found to be equally as protective as in younger patients [126]. Similarly, in the LIPSIA CONDITIONING trial, the combination of RIC and local ischaemic postconditioning was effective in both age groups—under and over 65 years old [36]. Similar results were obtained after the analysis of confounders of RIPre cardioprotection in patients undergoing coronary artery bypass grafting [80]. In a recent experimental study by Heinen et al., plasma of *young male volunteers*, subjected to RIC, reduced infarct size in isolated hearts from *aged rats* [49]. However, RIC plasma of *aged male volunteers* had no protective effect in *young rat hearts* [49]. This indicates that aging affects the RIC-induced release of a humoral factor, but not the susceptibility of myocardium to the protective effect of this phenomenon.

One of the main purposes for investigating the mechanisms of endogenous cardioprotective phenomena is the potential to develop pharmacological therapy or electronic devices that can fully mimic its positive effects. These could have the advantage of being able to be applied or administered significantly more quickly than the RIC procedure, which requires up to 40 min. As RIPre/RIPer cardioprotection is mediated by the activation of vagal efferents [5, 93], it is logical to assume that vagal nerve stimulation could potentially reproduce the effects of RIC. The obvious question arising here is whether monolateral vagal nerve stimulation can achieve this goal. In experimental studies, electrical stimulation of right [3, 18, 20, 72, 73], left [123, 124] or both vagal nerves [134] limited myocardial infarct size when started either before [18, 20, 72] or during [3, 73, 123, 124, 134] myocardial ischaemia, or at the onset of reperfusion [18]. In a clinical study by Yu et al., low-level transcutaneous stimulation of the vagal branch within the area of the right tragus, in patients presenting with STEMI, was followed by the reduction of the incidence of reperfusion-related ventricular arrhythmia, the area under curve for creatine kinase-MB and myoglobin over 72 h, and blood levels of inflammatory markers [52, 150]. In the study by Basalay et al., the activity of only one vagal nerve—either right or left—was sufficient and contributed equally to mediate cardioprotection established by RIPre. On the other hand, functional integrity of both nerves was required to establish cardioprotection when the remote conditioning stimulus was applied during myocardial ischaemia [6]. However, the differences in the mechanisms of RIPre and RIPer leading to this difference [6] have not been explained. Another arising question is—whether electrical vagal nerve stimulation can completely mimic all aspect of RIC, which include the indirect stimulation of the release of humoral factors [52].

While the importance of the activation of parasympathetic efferents in RIC-mediated cardioprotection is now unambiguous, the role of the *sympathetic* nervous system in this phenomenon has not been described clearly. However, it has been demonstrated that beta-blockers reduce infarct size in STEMI patients undergoing PCI [61], and this group of drugs is currently included in the guidelines for the management of these patients [60]. It has been shown that beta-adrenoreceptors are involved in the mechanisms of myocardial infarct-limiting effect of remote pre- and postconditioning of trauma [128, 129]. The existence of some common, inherent mechanisms, such as the activation of C sensory fibers and the K(ATP) channels for both remote preconditioning of trauma [65] and RIPre/RIPer [5, 83, 117], may be an argument to suggest that sympathetic beta-adrenergic activation is involved in the mechanisms of RIC cardioprotection. On the other hand, it has been demonstrated in rats that exercise training, which shares some molecular mechanisms with RIC [108, 121], augments the dynamic heart rate response to vagal but not sympathetic stimulation [97]. Moreover, it is known that sympathetic nervous response to ischaemia–reperfusion injury is altered with RIPre [84]. In the CONDI trial, the infarct-limiting effect of RIC was preserved in beta-blocker users [126]. Although there are no available data on the effect of beta-blockers on the efficacy of RIC in other clinical trials *with STEMI patients*, the fact that remote conditioning was able to reduce infarct size in the clinical study, where almost all the patients were taking beta-blockers [36], reinforced the CONDI results (Table 1). Similarly, medication with beta-blockers was not a significant confounder of RIPre cardioprotection in patients undergoing *cardiac surgery* [80]. In contrast, a meta-analysis of 15 clinical trials, including 1155 patients randomised to treatment with or without RIPre, showed an attenuated effect of this intervention *in cardiosurgery patients* on perioperative beta-blocker treatment [153].

**Table 1:** The use of beta1-blockers in clinical studies on the effect of remote ischaemic conditioning in patients undergoing primary percutaneous coronary interventions

First author	Year	Country	Number of patients	Beta-blockers use (RIC/control, %)	RIC effect	Endpoints
Bøtker HE	2010	Denmark	333	15/15	Yes	MSI (SPECT)
Rentoukas I	2010	Greece	96	99/100	n.s.	Peak TnI
Crimi G	2013	Italy	100	13/20	Yes	AUC CK-MB
Eitel I	2015	Germany	696	96/98	Yes	MSI (MRI)
Verouhis D	2016	Sweden	93	10/15	No	MSI (MRI)
Gaspar A	2018	Portugal	258	14/16	Yes	Progression of heart failure

*RIC* remote ischaemic conditioning, *MSI* myocardial salvage index, *SPECT* single-photon emission-computed tomography, *TnI* troponin I, *AUC* area under curve, *CK-MB* creatine kinase-muscle/brain, *MRI* magnetic resonance imaging

## Remote ischaemic conditioning of the brain

In addition to protecting the heart, RIC is potentially able to protect any other organ or tissue. It has been shown in a series of experimental studies that RIPre, RIPer, RIPost and delayed RIPost reduce brain infarct size in a rodent model of acute focal ischaemia/reperfusion brain injury [24]. Interestingly, the reperfusion ‘time-window’, during which delayed RIPost was effective, seems to be longer in an ischaemic stroke model [104, 114] than in a STEMI model [4, 5]. In this regard, in a rat model with 100-min focal brain ischaemia, RIPost was able to reduce infarct size when initiated within 30 min of reperfusion [104]. Impressively, when subject to 30-min brain ischaemia, RIPost was effective up to 3 h of reperfusion [114]. A meta-analysis of 13 randomized controlled trials, which included a total of 794 study participants who either suffered from, or were at risk from brain ischaemia and reperfusion injury, suggested that RIPost can offer cerebral protection for stroke patients [152]. Compared with controls, RIPost was shown to be able to reduce the recurrence of stroke or transient ischaemic attacks, levels of National Institutes of Health Stroke Scale score, modified Rankin Scale score and high-sensitivity C-reactive protein [152].

The studies on the mechanisms of RIC-induced neuroprotection are quite scarce in comparison with the amount of studies on the mechanisms of RIC-induced cardioprotection. Similar to RIC-induced cardioprotection, there is evidence for the involvement of the neural pathway in the mechanism of RIC of the brain [91, 104, 114, 139, 149], including the possible involvement of endogenous opioids [113, 154] and CGRP [113]. On the other hand, in one study, local electrical stimulation enhanced the neuroprotective effect of RIPost in a rat stroke model [145], which might suggest that the mechanisms underlying the therapeutic effects of these interventions are different. Pignataro et al. demonstrated that the neuronal isoform of nitric oxide synthase takes part in the neuroprotective effect of RIPost [104]. Further investigation of the neuroprotective mechanism of RIC is important to establish the most effective and safe implementation of this phenomenon to clinical practice.

The activity of the cardiovascular system is continually modulated by the central nervous system, ensuring coordination and regulation of regional cardiac electrical, mechanical and metabolic indexes throughout each cardiac cycle [2]. This fine regulation is provided by the neuronal elements, which are distributed from the level of the insular cortex to the intrinsic cardiac nervous system, and are in constant communication with one another [2]. In this regard, both ischaemic and haemorrhagic strokes can cause abnormalities in autonomic nervous system activity, followed by functional imbalance within the cardiovascular system, and in certain situations—even by irreversible cardiomyocyte damage, determined as serum troponin elevation [33]. On the other hand, nociceptive sensory inputs arising from the ischaemic heart represent a stimulus that can evoke discord within and among different levels of the hierarchy of the neuronal elements connecting heart, brain and central nervous system [2, 54]. Bearing in mind the existence of these bidirectional feedback interactions between the heart and brain [2], and specifically the involvement of brain subcortical structures in ‘remote preconditioning reflex’ [44, 93], more detailed understanding of the mechanisms of RIC-induced *neuroprotection* would shed more light on the mechanisms RIC-induced *cardioprotection* and myocardial ischaemia/reperfusion injury.

## Conclusions

Investigating the mechanisms of RIC is the essential step on the road to its translation to patient benefit, and specifically to the discovery of therapies or electronic devices, which would be able to mimic the beneficial effects of this phenomenon. Experimental and clinical data indicate that neural mechanisms, known as the ‘remote preconditioning reflex’, have an important contribution into RIC phenomena. However, there are still gaps in our understanding of the neural mechanism:

- Though the mechanism of RIPre has been extensively investigated, the mechanism of delayed RIPost is less clear. According to experimental data so far, delayed RIPost could be effective in patients with neural impairments, in whom the anti-ischaemic effect of RIPre is not expected.
- Although the involvement of vagal nerves in RIPre and RIPer is certain, an important question in terms of future drug discovery is whether these phenomena provide cardioprotection via the increase of ACh concentration in left ventricle myocardium.
- In terms of the future possibility to mimic or increase the effects of RIC using direct vagal nerve stimulation, a gap in the current knowledge is: why the effect of *monolateral* vagotomy is different for infarct-limiting effect of RIPre and RIPer.
- Sympathetic involvement in RIC is another gap in our understanding of these phenomena. The interaction of the sympathetic and parasympathetic nervous systems is fundamental and complex, so that, from a translational point of view, it would hardly be possible to modulate one of them without influencing the other. Notably, drugs modulating sympathetic activity are widely used in routine clinical practice—this fact is important to bear in mind when designing clinical studies.
- Revealing the mechanisms of RIC-induced *neuroprotection* is an important goal for future research not only to be able to use this phenomenon most efficiently in clinical practice, but also as it could help better understand the mechanisms of *cardioprotective* effects of these phenomena.
